# Aurora Kinase A: Integrating Insights into Cancer, Inflammation, and Infectious Diseases

**DOI:** 10.1080/29933935.2024.2419069

**Published:** 2024-10-24

**Authors:** Nidhi Varshney, Rajan Kumar Pandey, Amit Mishra, Sunil Kumar, Hem Chandra Jha

**Affiliations:** a Department of Biosciences and Biomedical Engineering, Indian Institute of Technology Indore, Indore, India; b Department of Medical Biochemistry and Biophysics, Karolinska Institute, Stockholm, Sweden; c Department of Bioscience & Bioengineering, Indian Institute of Technology Jodhpur, Karwar, Jodhpur District, Rajasthan, India; d Division of Agricultural Bioinformatics (CABIN), ICAR-Indian Agricultural Statistics Research Institute (IASRI), New Delhi, India

**Keywords:** Aurora Kinase A, cancer, virus, bacteria, infectious disease, inflammation

## Abstract

Aurora kinase A (AURKA) is crucial in regulating cell division and maintaining genomic stability, making it significant in cancer biology. This review explores AURKA’s structural and functional roles, emphasizing its involvement in cell cycle progression. Beyond cancer, AURKA emerges as a multifaceted player in host cell modulation, exerting influence over inflammatory responses, cell death mechanisms, and autophagy pathways. Moreover, recent research highlights its involvement in viral and bacterial infections. Numerous viruses, for their replication and assembly, modulate host cell cycle progression. Various viruses modulated AURKA to induce viral-mediated tumor. It also emerged as a key modulator in various gut microbiome-mediated cancers. Further, it also imparts oncogenic effect by modulation in cytoplasmic and mitochondrial regions of the cell. It hampers DNA replication independent of its kinase domain. Understanding AURKA’s diverse roles underscores its potential as a promising drug target, offering therapeutic avenues for various diseases. This comprehensive exploration navigates through complex landscape of AURKA biology, paving way for future investigations and therapeutic interventions.

## GRAPHICAL ABSTRACT

**Figure:**
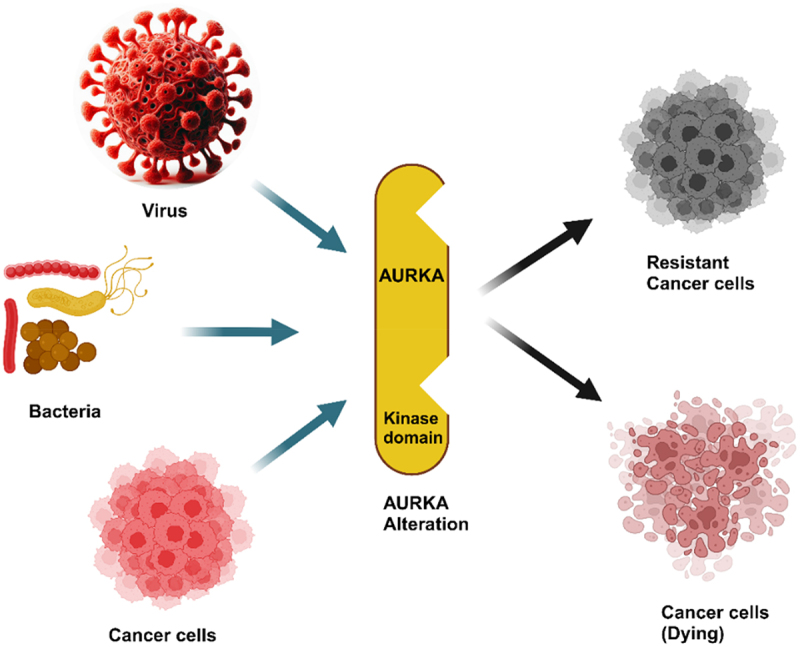

## Introduction

Aurora Kinase A (AURKA) is responsible for carrying out various essential processes of mitotic cell division, such as centrosome maturation, mitosis entry, mitotic spindle formation, and cytokinesis.^1^ Due to its potent regulatory roles on multiple signaling pathways, AURKA has been the focus of extensive research. The AURKA gene in humans is found on chromosome 20q13.^2^ The structure of AURKA comprises two domains, each playing a crucial role in orchestrating cellular life. It is localized on the centrosome by the N-terminal domain, which depends on microtubules. A degradation box (D-Box) and an activation loop are the two conserved domains in the catalytic region which belong to the C-terminal. Activation of AURKA is induced by phosphorylating in the highly conserved RxT motif of the activation loop at threonine residue. The D-Box degrades AURKA via the ubiquitin-mediated proteasome pathway.^3^ AURKA was initially identified as a mitotic kinase that engages centrosome and spindle activity during mitosis and phosphorylates particular substrates. Later research on tumor development revealed that it is an overexpressed oncogene and exhibits gene amplification in various human tumors.^4^ AURKA has also been found to have a variety of functions in controlling the growth and spread of tumors, including promoting cell division, triggering cell survival and anti-apoptosis/or necroptosis signaling, causing genomic instability, and influencing the cell migration, epithelial-mesenchymal transition (EMT) and cancer stem cells (CSCs).^3^ Recent research has revealed that novel cancer hallmarks linked to AURKA alteration regulate tumor progression and therapy resistance. Thus, AURKA serves as a molecular marker for cancer diagnosis and prognosis in addition to being a target for cancer treatment.

**Figure 1.**
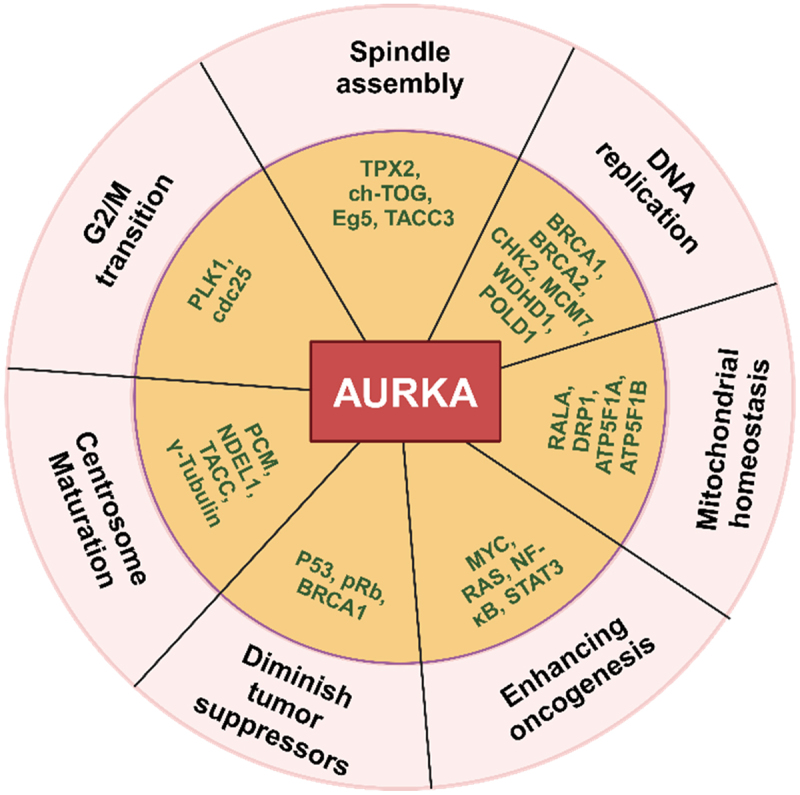
The multifunctionality of AURKA. The protein is associated with a diverse range of biological pathways (pink circled area), including G_2_/M phase transition, mitosis, mitochondrial homeostasis, DNA replication, and modulation of tumor suppressors and oncogenes via interactions or kinase-substrate relationships with its partnering proteins (yellow circled area).

AURKA is activated through phosphorylation and dephosphorylation, as well as through an autophosphorylation process. When AURKA is phosphorylated at Thr288 (and possibly Thr287), it activates downstream substrates. Conversely, when AURKA is auto-phosphorylated at Thr288, it increases kinase activity by phosphorylating Ser89.^5^ The phosphorylation of AURKA on its activation loop is necessary for AURKA activation.^5^ In human cancer, co-factors such as Bora,^6^ Ajuba,^7^ PPI-2 (protein phosphatase inhibitor-2),^8^ PAK1 (p21-activated kinase 1),^9^ and TPX2 (target protein for Xenopus kinesin-like protein 2)^10^ may activate the AURKA while PP2A (protein phosphatase 2A) may deactivate this kinase.^11^ In a manner resembling a positive feedback loop, AURKA appears to be activated by cyclin-dependent kinase 1 (Cdk1) and potassium channel tetramerisation domain containing 12 (KCTD12).^6,12,13^ Deubiquitination of AURKA may also be aided by ubiquitin-specific peptidase 3 (USP3).^14^ The microtubule-binding protein TPX2 is the most well-characterized AURKA regulator. Since TPX2 and AURKA are co-expressed during the cell cycle progression, the complex may have applications as a novel holoenzyme.^15^ Increased auto-phosphorylation and allosteric activation following binding to TPX2 were caused by mutations or overexpression of AURKA, which subsequently encouraged downstream pathways like oncogenic MYC.^16^ AURKA plays a crucial role in the p53 pathway, particularly in the checkpoint reaction that phosphorylates and stabilizes p53 to initiate the oncogenic transformation.^17^ AURKA may also phosphorylate other target proteins, such as TACC3 (transforming acidic coiled-coil protein 3), HDAC6 (histone deacetylase 6), and CDC25B (cell division cycle 25 homolog B).^1^ These targets have the potential to trigger several pathways, including NF-κB (nuclear factor kappa B) and STAT3 (signal transducer and activator of transcription 3), which in turn promote cell survival, inflammation, proliferation, angiogenesis, self-renewal, metastasis, and the development of tumor malignancy and resistance^1^ (Figure 1). The biological significance of AURKA as a cancer hallmark is demonstrated in this review. We examined AURKA’s function in autophagy, inflammation, and cell death. Furthermore, we have discussed the significance of AURKA in various bacterial and viral infection-induced diseases such as cancer. To the best of our knowledge, this is the first review in which AURKA was investigated in viral and bacterial-induced cancers. We also included the kinase-independent functions and combinational strategies that target AURKA in precision medicine for cancer treatment.

## AURKA and its associated signaling molecules in cancer

According to the Cancer Genome Atlas (TCGA) database, 33 cancer types exhibited amplification in AURKA expression. Tumor tissues, including the bladder, stomach, breast, liver, and lung, were shown to have higher levels of AURKA than adjacent normal tissues. This elevated level has been linked to poor prognosis and survival, clinical aggressiveness, and therapy resistance.^18^ As a result, the involvement of AURKA in cancer formation and therapeutic response warrants further investigation, indicating AURKA’s significant potential as a therapeutic target in cancer. AURKA may phosphorylate RAS-association domain family 1, isoform A (RASSF1A), an emerging tumor suppressor, disrupting RASSF1A-mediated microtubule stability and M-phase cell cycle arrest and causing an uncontrolled proliferation in malignancies.^19^ AURKA phosphorylates IκBα, a NF-κB inhibitor, activates it signaling pathway. AURKA can modulate the activity of p53, a widely recognized tumor suppressor, by phosphorylating it on both Ser215 and Ser315 residues.^20^ This phosphorylation hinders p53’s ability to transcribe genes and promotes the destruction of p53 by Mdm2.^21^

Patients with a poor prognosis are typically due to metastases caused by EMT. AURKA increases the expression of SLUG, an EMT transcription factor, and fibrillin 1 (FBN1), an essential fibrillin that regulates the microenvironment.^22^ AURKA inhibits the expression of E-cadherin and β-catenin, which regulate cell-cell adhesion and promotes EMT.^23^ AURKA also activates the Wnt and Akt signaling pathways simultaneously by lowering H3K4/H3K27 methylation on the Twist promoter, a well-known negative MET factor, and then promotes EMT.^24^ Other constitutive activation of oncogenic signaling pathways, including as Raf-1, Myc, and OCT4, promotes EMT progression by stabilizing and accumulating AURKA.^25^ Furthermore, AURKA overexpression may significantly elevate the expression of matrix metalloproteinases (MMP)-2, MMP-7, and MMP-10, resulting in the breakdown of extracellular matrix proteins and increased tumor cell motility and metastasis.^26^ Interestingly, overexpression of AURKA has been discovered in CSCs that exhibit self-renewal and differentiation into all cell types.^27^ AURKA activates Wnt signaling pathways in glioma-initiating cells (GIC) by interacting with AXIN and stabilizing β-catenin. This promotes GICs’ ability to self-renew. The β-catenin/TCF4 combination can activate AURKA and inhibit GSK3β, leading to increased β-catenin stability.^28^ As a result, the AURKA/Wnt signaling pathway creates a positive feedback loop that enhances the expression of core CSCs.^28^ AURKA in tumor cells engages with alternative oncogenic pathways, including Myc, PKC/MAPK, BCR/ABL, NFκβ, and Wnt/β-catenin pathways, to promote cell proliferation, survival, and resistance to therapy.^22^ Furthermore, AURKA plays a pivotal role in controlling the Pi3K/Akt pathway, which promotes malignant transformation and confers resistance to anti-cancer treatments.^29^

## AURKA in host cell modulation

### Inflammation

**Figure 2.**
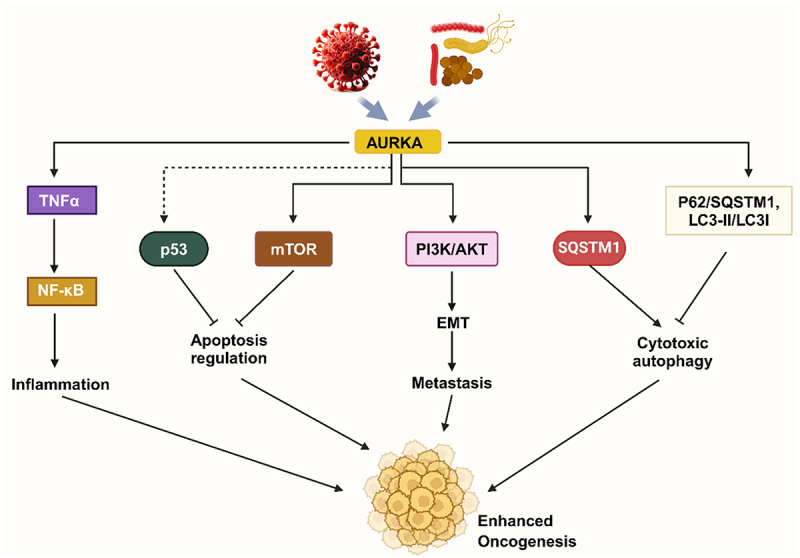
Role and mechanism of AURKA in viral and bacterial associated cancer in human. Viral and bacterial infections promote AURKA expression, which modulates various pathways and enhances oncogenesis by inducing inflammation and metastasis and blocking apoptosis and autophagy.

The role of AURKA is well established with cancer such as breast, gastric, lung, and liver cancer. However, its association with inflammation has been studied a little. Chronic inflammation and NF-κB activation are critical steps in several cancers^30^ showed that AURKA-enhanced expression correlates with higher chronic inflammatory scores.^30^ The AURKA overexpression significantly enhanced the NF-κB phosphorylation and activation. Without certain stimuli, NF-κB transcription factors are sequestered in the cytoplasm when bound to inhibitory IκB proteins. IκB can be phosphorylated by two similar protein kinases, IKKα and IKKβ. This is thought to be a convergence step for most signal transduction pathways that activate NF-κB. Moreover, AURKA phosphorylates and directly binds to the IκBα subunit, activating NF-κB. Further, the activation of NF-κB induced by TNF-α was eliminated by inhibiting AURKA, independent of IKKα/β. Thus, AURKA was found to be overexpressed during chronic inflammation to promote NF-κB activation and tumorigenesis^30^ (Figure 2).

Further, Tang et al. demonstrated that lesional psoriatic tissues had elevated levels of AURKA, and psoriasis tissue expressed more AURKA than psoriasis skin.^31^ AURKA knockdown increased autophagy while reducing keratinocyte inflammatory responses and AIM2 inflammasome activation. Thus, by preventing autophagy-mediated AIM2 inflammasome suppression, AURKA increased the inflammation associated with psoriasis diseases.^31^

### Resistance to cell death

#### Apoptosis

Chemotherapeutic drugs and radiation therapy primarily kill the cancer cells by inducing apoptosis. Apoptosis resistance was developed by numerous cancer cell types, resulting in drug resistance to cancer treatments.^32^ In triple-negative breast cancer (TNBC) cells, it has been discovered that AURKA stimulates mTOR to promote cell proliferation. AURKA inhibition significantly decreased the amount of p-mTOR in AURKA-induced TNBC cells, indicating that AURKA positively controls p-mTOR expression.^33^ The epigenetic study showed that the N-terminal region of AURKA was methylated by histone methyltransferase multiple myeloma SET domain protein (MMSET) at the K14 and K117 methylation sites.^34^ Through proteasomal degradation, AURKA methylation decreased the stability of p53 while promoting cell division and inhibiting apoptosis. Furthermore, alisertib’s inhibition of AURKA activity triggered calpain signaling and caused gastric cancer cells to undergo apoptosis by cleaving Bax and p27^35^ (Figure 2). Alisertib also blocks the cell cycle in human epithelial ovarian cancer cells by G2/M arrest, induces apoptosis, and inhibits EMT via phosphatidylinositol 3-kinase/Akt/mTOR-mediated and sirtuin 1-mediated pathways.^36^ Alisertib treatment activates caspase-2, which induces apoptosis by cleaving Bid into truncated Bid, a suppressor of both Bcl-xL and Mcl-1. This suggests a critical role for the Bcl-2 protein network in AURKA inhibitor-induced apoptosis and implies that BH3-mimetics that target Bcl-xL could aid in overcoming cancer cells’ resistance to AURKA inhibitors.^37^ Further, AURKA inhibitor TCS7010 significantly reduced Ewing sarcoma growth *in vitro* and *in vivo* by inducing ferroptosis and apoptosis. AURKA is considered an endogenous repressor for ferroptosis and apoptosis by phosphorylation of NPM1 at the Thr95 position, thus stabilizing YAP1.^38^ Treatment with TCS7010 induces a time-dependent cleavage of caspase-2, caspase-7, and poly (ADP-ribose) polymerase (PARP) in colon cancer cells. TCS7010 activates the ROS-mediated UPR signaling pathway in colon cancer cells, causing apoptosis.^39^

#### Necroptosis

In pancreatic cancer, AURKA directly interacts with receptor-interacting serine/threonine kinase (RIPK) 1 or RIPK3 to induce necroptosis as a local inhibitor.^40^ Glycogen synthase kinase 3β (GSK3β) phosphorylation at serine residue by AURKA, RIPK3, and the mixed lineage kinase domain-like (MLKL) complex formation was disrupted, necrosome activation was decreased, and necroptosis was hampered. The alteration in AURKA consequently led to resistance against necroptosis-mediated apoptosis.^40^

### Autophagy

As per the literature, autophagy plays a crucial role in chemoresistance.^41^ By inducing cytotoxic autophagy, chemotherapy drugs improve cellular apoptosis and drug sensitivity. The expression of the autophagy-associated protein SQSTM1 in breast cancer was found to be correlated with AURKA. Inhibition of AURKA decreases SQSTM1 and triggers cytotoxic autophagy^42^ (Figure 2). Pharmacological autophagy inhibitors (BAF or chloroquine) also increased cytotoxicity through AURKA depletion. Furthermore, p62/SQSTM1 and LC3B expression were markedly increased with alisertib treatment, which also promoted cytotoxic autophagy. When siRNA was used to deplete AURKA in pancreatic cancer cells, autophagic cell death resulted, along with an increase in the p62/SQSTM1 ratio and the LC3-II/LC3-I ratio.^43^ Moreover, AURKA prevented autophagy in lung cancer by preserving the transcription co-factor YAP’s protein stability.^44^ It has been established that AURKA controls autophagy via FOXO3a. The apoptosis of adipose-derived stem cells (ADSCs) induced by high glucose was inhibited by AURKA-mediated autophagy. ADSCs overexpressing cells showed AURKA decreased ADSC apoptosis and enhanced skin wound healing in diabetic mice.^45^ Furthermore, the infiltration of immune cells and the expression levels of genes linked to autophagy were correlated with the AURKA expression level. Nasopharyngeal carcinoma (NPC) cell migration and proliferation were also impeded by AURKA silencing.^46^

## AURKA in viral infections

Chronic viral infections can cause various illnesses, including cancer, which can significantly diminish a patient’s quality of life and increase their risk of death. Pathways such as regulating cell cycle progression, and evasion from cell death, known to cause neoplasia in tumor cells, are also the targets of viruses that cause oncogenesis. It’s interesting to note that viruses introduce comparable metabolic modifications into the host cells, and more significantly, it appears that these modifications are necessary for the viruses’ endurance and amplification. For example, the human papillomavirus, hepatitis B and C viruses, Epstein-Barr virus, and Kaposi’s Sarcoma-associated virus modify the central carbon metabolism, frequently altered in tumor cells. Further, viruses manipulate cell cycle progression to facilitate resources and cellular conditions favorable for viral assembly and replication. The upcoming section will be discussed about the relationship between the viruses and AURKA. However, the precise mechanism driving this association still needs to be discovered.

### Epstein-Barr virus

The Epstein–Barr virus (EBV) infects most people, causing chronic infections that are typically silent.^47^ Furthermore, it is responsible for more than 200,000 cancer cases annually, the majority of which are lymphomas, including Burkitt’s lymphomas (BL), nasopharyngeal carcinoma (NPC), gastric carcinoma (GC), and lymphoproliferative disease following transplantation.^48–50^ Although EBV latency proteins promote growth, their lytic gene products have been connected to the development of tumors. Viral noncoding RNA EBER2 increases the expression of UCHL1 deubiquitinase, increasing the expression of cyclin B1 and AURKA and accelerating the growth of B cells.^51^ EBV’s early lytic gene product BGLF4 affects the cellular kinases AURKA, AURKB, ATR, and ATM activity.^52^ Additionally, the latent antigen of EBV, EBNA1, which is expressed throughout the virus’s latency stage, interacts with AURKA to accelerate the development of gastric cancer. AURKA knockdown decreases cell migration and proliferation and induces apoptosis in EBV-infected gastric cancer cells through the cleavage of Casp 3, 9, and PARP1.^53^ The gene cluster enhanced by EBV infection also includes CCNA2, CCNB1/2, CDK6, CDC2/3/5/7/8, AURKA/B, MYB, BATF, and IRF4. After two days of infection, the expression of these genes peaked on day four and remained at that level after that.^54^

### Kaposi’s Sarcoma-associated herpesvirus (KSHV)

KSHV, also known as human herpesvirus 8, belongs to the gamma-herpesvirus family, which is linked to primary effusion lymphoma (PEL), multicentric Castleman’s disease (MCD), and Kaposi’s sarcoma (KS).^55–57^ One of the crucial latent antigens encoded by KSHV, the latency-associated nuclear antigen (LANA), expressed in virus-infected tumor cells that cause KSHV-associated cancers.^58^ In KSHV-associated human cancers, LANA can destabilize the p53 tumor suppressor’s functions and increase the expression of the cellular oncogene AURKA.^55^ Zhu et al. have shown that co-immunoprecipitation assay depicts the physical interaction between LANA and AURKA.^59^ Depletion of APC3, an essential core protein of APC/C, increased the substrate level of APC/C (AURKA, AURKB, CCNB1, CCNA2, GMNN, securin, and PLK1) in both KSHV-Lyt-infected LECs and iSLK.219 cells.^60^ KSHV+ tumor cell survival in PEL disease is correlated with sphingolipid metabolism. AURKA and CDCA3, two novel cellular genes regulated by sphingolipid metabolism and necessary for PEL survival.^61^

### Hepatitis C virus

Chronic hepatitis C virus (HCV) infection is a significant factor in the development of liver fibrosis, cirrhosis, and hepatocellular carcinoma (HCC).^62^ The primary constituent of the viral nucleocapsid, the HCV core protein, has been linked to several processes, including, cell-cycle modulation, cellular proliferation and transformation.^63,64^ TCGA and HPA data showed 63 upregulated/hypomethylated genes and 122 downregulated/hypermethylated genes in HCV-positive patients such as AURKA, CDKN3, etc. Moreover, FOS and abnormally methylated AURKA may be helpful therapeutic targets for managing HCV-positive HCC.^65^

### Human cytomegalovirus

Human cytomegalovirus (HCMV) is a highly host-specific herpesvirus that is widely distributed and can cause severe, occasionally fatal infections in immunocompromised people, including recipients of bone marrow allograft transplants and patients with AIDS.^66–68^ Various studies have shown that gene expression analysis of cell cycle markers (AURKA, AURKB, NEK6) is downregulated in HCMV-infected samples compared to uninfected samples.^68,69^

### Hepatitis B virus

The main risk factor linked to a sharp rise in the incidence of hepatocellular carcinoma (HCC) is chronic, persistent hepatitis B virus (HBV) infection.^70^ It has been shown that HBV DNA may integrate into the host genome and that the integration site may be found in the area controlling cell division and proliferation. This could considerably raise the risk of oncogenesis.^71^ A study by Xie et al. showed that the expression of nine hub genes was significantly linked with poor overall survival of HBV-infected patients. These genes were AURKA, FOXM1, CDC45, ZWINT, PBK, CDK1, NDC80, TYMS, and TPX2.^72^ These nine essential genes may be exploited as novel targets for HBV+ HCC therapy and prognostic indicators.

### Human immunodeficiency virus

Human immunodeficiency virus type 1 (HIV-1) is a virus that spreads effectively when it comes into direct contact with cells that are either infected or not through a structure called a virological synapse (VS).^73,74^ HIV activity in cell-to-cell fusion and transmission was markedly enhanced when the important mitotic regulator Aurora kinase B (AURKB) in HIV-1 infected cells was inhibited; however, cell-free infection was not much affected.^75^ Greenwood et al. found that activation of aurora kinase A (AURKA) and B (AURKB) in cells infected with WT but not ∆Vif viruses.^76^

### Severe acquired respiratory syndrome-corona virus − 2 (SARS-COV2)

About 3.5 million people have died globally as a result of SARS-CoV-2, the virus causing the current severe coronavirus disease 2019 (COVID-19) pandemic, in less than 18 months.^77,78^ The molecular mechanisms underlying SARS-CoV-2’s capacity to navigate different body organs and modify cellular environments remain unclear.^79^ SARS-CoV-2 infection was associated with significantly higher expression of AURKA at 12 hpi, which decreased at 24 hpi and then increased again at 48 hpi, suggesting that the infection may impede the host cell cycle progression.^80^ Various studies have stated that numerous proteins involved in cell cycle regulation, including TOB2, AURKA, and AURKB, were reduced upon SARS-CoV-2 infection.^81^ Xing and colleagues have demonstrated a strong correlation between the expression of ChoMCyto genes and the severity of COVID-19 patients and anti-SARS-CoV-2 activity *in vitro*. Among these genes, TP53 interacts with the greatest number (INSIG1, MYBL2, AURKA, CCNB1, and STMN1). Furthermore, SARS-CoV-2 compounds also target key cellular pathways and downregulate 30 important genes such as AURKA, CCNB1, etc.^82^

## AURKA in bacterial infections

Cancer has been connected to several bacterial infections, thought to be responsible for 20% or so of cancer cases.^83^ Although precise mechanisms underlying these findings remain disputed, research into the mechanistic connections between infection and cancer development is still ongoing. Bacterial dysbiosis alters the host cells, initiates inflammation, and ultimately tumorigenesis. Oncogenic modulators of different bacteria also halt the cell cycle checkpoints and lead to cancer. This review highlights some of these bacteria and their association with AURKA in causing cancer. However, the underlying mechanism of the association is still unknown.

### *Helicobacter pylori* infection

*Helicobacter pylori* (*H. pylori*) infection is thought to affect more than half of the world’s population, indicating a very high prevalence of infection.^84–86^ According to epidemiological and genomic research, strains expressing CagA may cause infection, leading to GC development.^87–89^ AURKA is overexpressed in about 60% of gastric cancer cases.^85^ AURKA promotes the proliferation and survival of cancer cells in response to *H. pylori* infection. AURKA expression significantly increases in gastric cancer patients with *H. pylori* infection than in those without the infection.^90^ According to another study, in GC cells, *H. pylori*-induced expression of SOX9 and LGR5 is reduced by AURKA knockdown. This suggests a functional signaling axis connects AURKA and *H. pylori* downstream effects.^91^ Gomaa et al.^92^ demonstrated that AURKA amplifies EIF4E-cap-dependent translation in response to *H. pylori* infection. Moreover, there is a correlation between increased expressions of AURKA and SOX9 in samples of human gastric cancer.^92^ Another study found that AURKA phosphorylates RPS6KB1, which enhances cell proliferation, survival, and the formation of xenograft tumors in mice.^93^ Thus, AURKA can be used as a biomarker in treating *H. pylori-*associated gastric cancer.

### Staphylococcus aureus

*Staphylococcus aureus*, also known as *S. aureus*, is a conditional pathogen usually present in mammals’ intestines but can also infect other organs, particularly the skin, nose, and throat.^94^ Yadav et al. have shown that *S. aureus* infection was activated by AURKA-responsive WNT signaling, which triggered septic arthritis, resulting in synovium inflammation.^95^ Various genes are downregulated in *S. aureus* infection, including AURKA, BUB1, BRCA1, etc.^96^

### Salmonella typhi

Salmonella is a gram-negative enteric pathogen with over 2500 serovars and can infect various hosts.^97^ Non-Typhoidal *Salmonella* (NTS) cause significant chronic infections from a clinical and epidemiological standpoint, with rates of 3.9% in children and 0.15% in adults.^98^ AURKA, BUB1, and other genes related to the cell cycle are regulated by *S. typhi* infection. Since these genes are typically only accumulated during a particular cell cycle stage, the downregulation of all G1/S, S, G2, and G2/M transcription programs suggests that some of the infected mucosal cells are not replicating.^99^

### Mycobacterium tuberculosis

The principal cause of tuberculosis (TB) in humans is *M. tuberculosis* (Mtb), a member of the Mycobacterium tuberculosis complex (MtbC).^100,101^ Some Mtb antigens modulate cell death as a survival mechanism by blocking apoptosis and activating other cell death pathways, like necrosis, that facilitate its spread.^102^ To prevent this necrosis, AURKA physically interacts with RIPK1 and RIPK3.^101,103^ Several genes related to the cell cycle (AURKA, CDC25, BIRC5, etc) that were elevated in lung cancer were also altered in Mtb infection.^104^

## AURKA inhibitors and their clinical trials

So far, numerous AURKA-specific or pan-aurora kinase inhibitors have been developed, out of which, 11 complete at least one clinical study for cancer patients (Table 1). Out of the 11 drugs now being studied in clinical trials, alisertib (MLN8237), is AURKA inhibitors that specifically target AURKA 200-fold more than AURKB. Alisertib has been intensively researched as an inhibitor of AURKA in various types of tumors, such as lymphoma, small cell lung cancer, ovarian cancer, leukemia, gliomas, and myeloma.^18^ The findings from the phase 1 and 2 trials suggest that alisertib demonstrates a tolerable toxicity profile in human subjects. Alisertib causes dose-limiting CNS toxicities at doses over 60 mg. However, these toxicities can be minimized by dividing a single dose into two doses taken twice daily.^115^ Prior clinical trials of alisertib focused on hematologic malignancies. An alisertib multicenter phase 2 clinical trial shown promising antitumor efficacy in patients with relapsed and refractory peripheral T cell lymphoma (R/R-PTCL).^116^ Patients with relapsed/refractory peripheral T-cell lymphoma (R/R-PTCL) who received treatment with Alisertib, after undergoing extensive prior treatment with conventional chemotherapy or targeted treatments, demonstrated an overall response rate of 30% (7% achieved complete response and 23% achieved partial response). Alisertib’s promising anticancer effects in hematologic cancers prompted a worldwide phase 3 clinical trial for the same type of malignancy. The results of the phase 3 trial revealed that alisertib had anticancer activity in patients with relapsed or refractory peripheral T-cell lymphoma (R/R-PTCL). However, it did not demonstrate any clear superiority compared to the other tested comparators (pralatrexate, romidepsin, and gemcitabine) in the treatment groups.^106^

**Table 1.** Aurora kinase inhibitors and their clinical trial status.

Drug Name	Targets	Cancer types	Completed clinical trials	NTC number	Title	Reference
Alisertib (MLN8237)	AURKA	Advanced solid tumors and lymphoma	Phase I	NCT01898078	An Open-Label, Phase 1, Two-Way, Cross-Over Study of the Effect of the Food on the Pharmacokinetics of MLN8237 (Alisertib) in Patients With Advanced Solid Tumors or Lymphomas	
		Advanced solid tumors or colorectal cancer	Phase I	NCT00500903	An Open-Label, Dose Escalation Phase 1 Study of MLN8237, a Novel Aurora A Kinase Inhibitor, in Patients With Advanced Solid Tumors	
		Head and neck cancer	Phase I	NCT01540682	Phase I Study of MLN8237 in Combination With Cetuximab and Definitive Radiation in Patients With Locoregionally Advanced Squamous Cell Carcinoma of the Head and Neck	
		Refractory multiple myeloma	Phase I/II	NCT01034553	Phase I/II Study of Combination of Aurora Kinase Inhibitor MLN8237 and Bortezomib in Relapsed or Refractory Multiple Myeloma	
		Relapsed aggressive B-cell lymphoma	Phase I/II	NCT01397825	A Multicenter, Phase 1-2 Study of MLN8237, an Oral Aurora A Kinase Inhibitor, in Patients With Relapsed or Refractory Aggressive B-Cell Lymphoma Treated With Rituximab and Vincristine	105
		Metastatic castrate-resistant prostate cancer	Phase I	NCT01094288	A Phase 1 Study of MLN8237, an Aurora A Kinase Inhibitor, in Patients With Advanced Solid Tumors Including Castration-Resistant Prostate Cancer Receiving a Standard Docetaxel Regimen	
		Relapsed/refractory peripheral T cell lymphoma	Phase III	NCT01482962	A Phase 3, Randomized, Two-Arm, Open-Label, Multicenter, International Trial of Alisertib (MLN8237) or Investigator’s Choice (Selected Single Agent) in Patients With Relapsed or Refractory Peripheral T-Cell Lymphoma	106
ENMD-2076	AURKA, FLT3, Src, VEGFR2, FGFR1	Multiple myeloma	Phase I	NCT00806065	A Phase 1 Study of ENMD-2076 in Patients With Relapsed or Refractory Multiple Myeloma	
		Relapsed hematological malignancies	Phase I	NCT00904787	A Phase 1 Study of ENMD-2076 in Patients With Relapsed or Refractory Hematological Malignancies	
		Triple-negative breast cancer	Phase II	NCT01639248	A Phase II Study of the Aurora and Angiogenic Kinase Inhibitor ENMD-2076 in Previously Treated Locally Advanced and Metastatic Triple-Negative Breast Cancer	107
		Advanced/metastatic soft-tissue sarcomas	Phase II	NCT01719744	A Phase II Study of Oral ENMD-2076 Administered to Patients With Advanced/Metastatic Soft Tissue Sarcoma	
		Advanced fibrolamellar carcinoma	Phase II	NCT02234986	A Phase 2, Multi-center, Open-label Study of Oral ENMD-2076 for the Treatment of Patients With Advanced Fibrolamellar Carcinoma (FLC)	108
AS703569 (MSC1992371A, Cenisertib)	AURKA, AURKB, ABL1, AKT, STAT5, FLT3	Pancreatic cancer	Phase I	NCT01097512	A Phase I, Dose-escalation Study of a Combination AS703569 and Gemcitabine Given to Subjects With Advanced Malignancies	
PHA-739358 (Danusertib)	Pan-Aurora kinase, ABL, TrkA, RET, FGFR1	Metastatic hormone-refractory prostate cancer	Phase II	NCT00766324	A Phase II Study of PHA-739358 in Patients With Metastatic Hormone Refractory Prostate Cancer	
AT9283	AURKA, AURKB, JAK2, JAK3, ABL(T315I)	Advanced non-Hodgkin’s lymphoma	Phase I	NCT00443976	A Phase I Study of AT9283 Given As a 24 hour Infusion on Days 1 and 8 Every Three Weeks in Patients With Advanced Incurable Malignancy	109
		Leukemia	Phase I	NCT00522990	A Phase I/IIa Open-label Study to Assess the Safety, Tolerability and Preliminary Efficacy of AT9283, a Small Molecule Inhibitor of Aurora Kinases, in Patients With Refractory Hematological Malignancies	110
		Multiple myeloma	Phase II	NCT01145989	A Phase II Study of AT9283 in Patients With Relapsed or Refractory Multiple Myeloma	111
AMG900	Pan-Aurora kinase	Acute myeloid leukemia	Phase I	NCT01380756	A Phase 1 Study Evaluating the Safety, Tolerability, Pharmacokinetics and Pharmacodynamics of Orally Administered AMG 900 in Adult Subjects With Acute Myeloid Leukemia	112
		Advanced solid tumors	Phase I	NCT00858377	A Phase 1, First-in-Human Study Evaluating the Safety, Tolerability, Pharmacokinetics and Pharmacodynamics of Orally Administered AMG 900 in Adult Subjects With Advanced Solid Tumors	113
BI-847325	AURKA, AURKC, MEK1/2	Neoplasms	Phase I	NCT01324830	An Open Label Phase Ia/Ib Study of Two Dosing Schedules of BI 847,325, Orally Administered Once a Day in Patients With Advanced Solid Tumours, With Repeated Cyclic Administration in Patients With Clinical Benefit	
PF-03814735	AURKA, AURKC	Advanced solid tumors	Phase I	NCT00424632	A Phase 1, Open Label, Multi-Center, Accelerated Dose-Escalation, Pharmacokinetic And Pharmacodynamic Trial Of The Oral Single Agent Aurora Kinase Inhibitor PF-03814735 In Patients With Advanced Solid Tumors For Whom No Standard Therapy Is Available	114
SNS-314	Pan-Aurora kinase	Advanced solid tumors	Phase I	NCT00519662	Phase 1 Open-Label Multicenter, Dose-Escalating, Clinical Study of the Safety, Tolerability, and Pharmacokinetic and Pharmacodynamic Profiles of SNS-314, a Novel Aurora Kinase Inhibitor, Administered to Patients With Advanced Solid Tumors	

Pan-aurora kinase inhibitors exhibit a broad range of targets, which include AURKA, AURKB, AURKC, FLT3, BCR-ABL, JNK, JAK2, and JAK3 kinases. However, they demonstrate a greater degree of specificity toward aurora kinases. While AURKA-specific inhibitors have a lower probability of causing adverse effects compared to pan-aurora kinase inhibitors, the latter provide some defense against the emergence of drug resistance in cells. So far, a total of 8 drugs that inhibit pan-aurora kinase, namely tozasertib, AMG900, AS703569, BI-847325, PF-03814735, SNS-314, AT9283, and danusertib, have been tested in at least one completed clinical trial for the treatment of cancer (Table 1). According to the findings from phase 1 and 2 clinical studies, these inhibitors were usually well tolerated with acceptable toxicities. However, their effectiveness as standalone treatments differed depending on the specific molecular subtype of the tumor.^18^

## Pharmacological combination strategy for targeting AURKA

**Figure 3.**
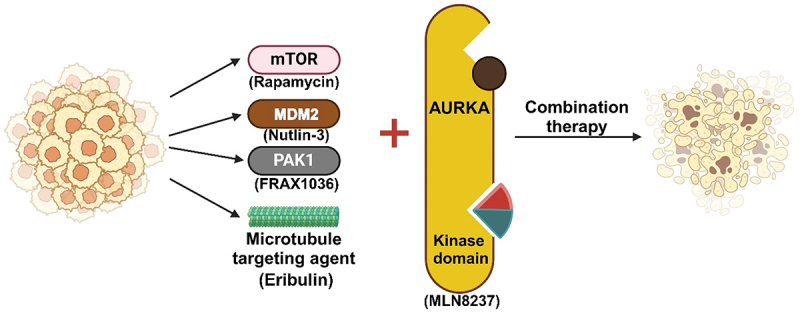
Pharmacological combination strategy for targeting AURKA. A combination of AURKA inhibitors and other therapeutics agents, including mTOR inhibitor, PAK1 inhibitor, MDM2 inhibitor, and microtubule inhibitor, have been shown to have synergistic effects against cancer cells.

Cancer cells modulate various molecules to inhibit the growth of these cells; the combinational strategy of AURKA inhibitors and other conventional cancer therapeutic agents showed a synergistic effect. For instance, the Bcl-2 inhibitor navitoclax, in combination with AURKA suppression, resulted in a significant reduction in the G_2_/M phase, an increase in the sub-G1 fraction, and the induction of apoptosis in the cells.^117^ Specific immune checkpoint inhibitors or small molecular kinase inhibitors have recently shown a synergistic anticancer effect when sensitized with AURKA inhibitors. Here, we highlight the potential synergistic approach of targeting AURKA with mTOR, PAK1, MDM2, and microtubule inhibitors (Figure 3).

### mTOR inhibitors

MTOR inhibitors solely caused G1 phase arrest. Researchers discovered that in TNBC, AURKA could control mTOR activity via the ERK1/2 pathway.^33^ Interestingly, synergistic effects were observed only when alisertib was given before rapamycin treatment, highlighting the significance of timing sequences in treatment regimens. It has been found that the anticancer effects of mTOR and AURKA inhibitors together work better than when either inhibitor is used alone.^118^

### Microtubule dynamics inhibitor

Eribulin, an inhibitor of microtubule dynamics, causes active AURKA to accumulate in TNBC and offers a novel idea for combination therapy. The suppression of AURKA led to cytotoxic autophagy through activation of the LC3B/P62 axis, which eliminated metastases but had no effect on the growth of breast cancers.^119^ Remarkably, alisertib plus eribulin induces a synergistic response that includes cytotoxic autophagy and apoptosis in breast cancers.

### PAK1 inhibitor

The high-level expressions of oncoproteins supplied an additional method for drug combination. When alisertib and FRAX1036, a highly selective PAK1 inhibitor, are combined, they have remarkably synergistic antitumor effects. For example, p21-activated kinase 1 (PAK1) and AURKA were frequently overexpressed in breast cancers.^9^ Alisertib plus FRAX1036 produced an exceptional therapeutic response in drug-resistant subclones, although drug resistance developed to single-agent FRAX1036. These combinations, based on various mechanisms and the ones that inhibited ERα phosphorylation and c-MYC expression, were also involved, in addition to PAK1.^120^

### MDM2 inhibitors

MDM2 antagonists and AURKA may activate p53 and stop the growth of tumors. NK cells, macrophages, and antigen-presenting dendritic cells were among the host immune cells that were further encouraged to infiltrate the tumor by the combination therapy of alisertib and (–)-nutlin-3.^121^ The other study demonstrated that p53-dependent postmitotic checkpoints were activated at the pseudo-G1 phase by a combination of MK-0457 and Nutlin-3 treatment and caused mitochondrial apoptosis and proapoptotic p53 signaling in AML.^122^

## Kinase independent function of AURKA

**Figure 4.**
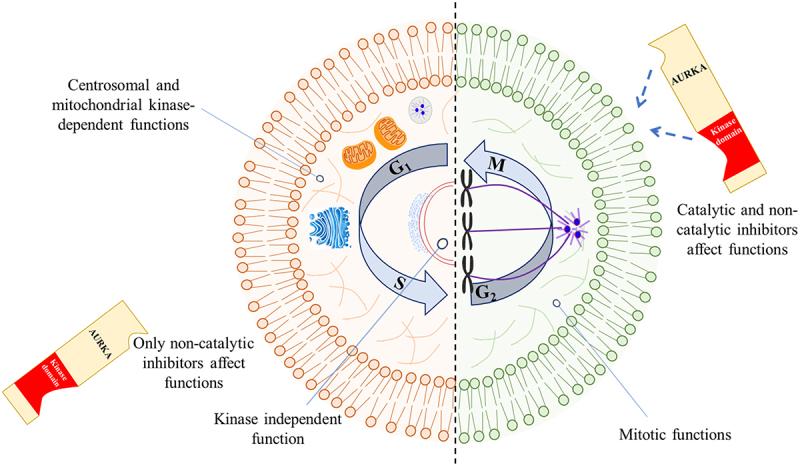
Targeting AURKA kinase-dependent and independent functions. The schematization of AURKA functions in cells reveals that these functions rely on either kinase activity or specifically localized AURKA pools (kinase independent activity). Thus, distinct AURKA function classes may be affected differently by particular classes of AURKA inhibitors.

In multiple malignancies, it has been observed that AURKA expression increases during the G_2_ and M phases of the cell cycle. On the other hand, new data indicates that AURKA is also expressed in non-cycling cells and during the G_1_ and S phases of the cell cycle. It has been linked to several non-mitotic processes,^123^ such as regulating the expression of the DNA damage-response genes BRCA1, CHK2, or BRCA2,^124^ protecting DNA forks during replication stress,^125^ disassembling primary cilia during cell cycle entry,^126^ and controlling mitochondrial dynamics and energy production.^127^ The kinase-independent role of AURKA in initiating DNA replication, which a class of allosteric inhibitors can inhibit, provides new opportunities for cancer treatment. AURKA and the replisome components MCM7, WDHD1, and POLD1 formed a multiprotein complex during G1, demonstrating that allosteric inhibitors, but not catalytic ones, block functional replisomes’ chromatin assembly. Allosteric inhibitors of AURKA, rather than catalytic inhibitors, increase the sensitivity of cancer cells to CDC7 kinase inhibition, a subunit of the replication-initiating factor DDK.^128^ Besides, AURKA is found in mitochondria of various human cancer cell lines and is imported there. Mitochondrial AURKA impacts both the production of energy and the dynamics of the mitochondria. ATP5F1A and ATP5F1B, two mitochondrial Complex V core subunits, interact functionally with AURKA. G0/G1 arrest can be initiated by modifying the AURKA/ATP5F1A/ATP5F1B nexus, which reduces glycolysis and mitochondrial respiration rates.^129^ Moreover, AURKA causes mitochondrial fragmentation independently, independent of RALA, when expressed at endogenous levels during interphase. AURKA, on the other hand, is overexpressed and increases mitochondrial fusion and ATP production. Researchers have proven that AURKA directly controls the activities of mitochondria, and the over-expression of AURKA increases mitochondrial interconnectivity, which in turn drives metabolic reprogramming.^127^ Furthermore, by interacting with essential elements of the autophagy pathway, AURKA causes the degradation of matrix/internal mitochondrial membrane proteins. The kinase, MAP1LC3, and the mitophagy receptor PHB2 form a tripartite complex on the inner mitochondrial membrane that initiates mitophagy independently of PARK2/Parkin. PHB2 must be phosphorylated on Ser39 for MAP1LC3 to interact with PHB2, which causes the tripartite complex to form.^130^ Therefore, the fact that the mitochondrial network is responsive to AURKA levels in various cell types emphasizes how crucial it is to clarify AURKA’s non-mitotic functions to thoroughly comprehend how it affects cancer and cell proliferation (Figure 4).

## Other important molecules for cancer

AURKA is a vital therapeutic agent for the treatment of cancer and its related diseases. Additional compounds may be incorporated alongside AURKA to enhance understanding of cancer therapies. The subsequent section provides a brief discussion of such compounds.

### Toll-like receptors (TLRs)

Toll-like receptors (TLRs) are pattern recognition receptors (PRRs) that significantly influence outcomes in several infectious and noninfectious disorders in humans, including cancer.^131,132^ TLRs function as innate immune receptors that specifically bind to pathogenic ligands, referred to as PAMPs, to initiate an innate immune response through the activation of the inflammatory signaling cascade.^132^ To date, 10 Toll-like receptors (TLRs 1–10) have been identified in humans, categorized into two groups: extracellular or cell surface TLRs (TLR1, TLR2, TLR4, TLR5, TLR6, and TLR11) and intracellular TLRs (TLR3, TLR7, TLR8, and TLR9). Upon stimulation, TLR binding facilitates the production of several adaptor proteins and downstream kinases, resulting in the induction of multiple signaling molecules, including essential pro-inflammatory mediators.^133^ TLRs have an important role in the genesis and proliferation of malignant tumors, as well as cancer prognosis. They facilitate carcinogenesis by releasing proinflammatory cytokines and anti-apoptotic chemicals, recruiting immune cells, and promoting cell proliferation inside the tumor microenvironment (TME) to establish a tumor-supportive environment.^133^ Furthermore, TLRs are linked to angiogenesis, metastasis, chemoresistance, and diminished survival rates. The detection of bacterial LPS activates TLR signaling pathways, leading to hyperinflammation that contributes to the development of carcinomas generated by bacterial infections, such as gastric, colorectal, and lung malignancies.^134^ Prior studies have demonstrated that stomach mucosal immunity responds to *H. pylori* infection by upregulating the expression of TLRs, hence creating an inflammatory milieu.^135^ Their involvement is crucial for the identification of *H. pylori* and the ensuing innate and adaptive immune responses against this bacterium. Consequently, advancements in immunobiological research indicate that TLRs may serve as viable targets for therapeutic intervention in inflammation-related diseases, autoimmune disorders, microbial infections, and human malignancies.

### Interleukins and other cytokines

Cytokines, as non-structural tiny proteins, are classified into several categories, including chemokines, interferons (IFNs), interleukins (ILs), tumor necrosis factors (TNFs), and lymphokines.^136^ Cytokines provide crucial functions as regulatory proteins in inflammatory immune responses and repair mechanisms by modulating both the innate and adaptive immune systems. Cytokines are produced by both immune and nonimmune, as well as hematopoietic and non-hematopoietic cancer cells.^137^ ILs are recognized as second messenger immunological glycoprotein macromolecules, comprising a subset of mammalian cytokines.^138^ Interleukins (ILs) engage in both pro-inflammatory and anti-inflammatory responses through their interaction with various receptors, such as Toll-like receptors (TLRs), which are implicated in damage-associated molecular patterns (DAMPs), microbial-associated molecular patterns (MAMPs), pathogen-associated molecular patterns (PAMPs), and xenobiotic-associated molecular patterns (XAMPs). The cytosolic portion of TLRs, which mediates intracellular signaling pathways, consists of a Toll/IL-1 receptor (Toll/IL-1 R) (TIR) domain. This domain is prevalent and uniform among TLRs, with the interaction of ILs uniting them as a cohesive structure.^139^ Conversely, ILs and TLRs play a role in homeostasis, cancer, and autoimmune and infectious disorders.^131,140^ Moreover, additional research indicated that MLN8237 (an AURKA inhibitor) prompted cellular senescence in LPS cell lines, evidenced by elevated levels of DcR2, a senescence biomarker, and enhanced expression of cytokines linked to the senescence-associated secretory phenotype, such as interleukin (IL)‑1α, IL‑6, and IL‑8.^141^ Expression of IL‐2, IFN‐γ, and perforin increased in AURKA-silenced cells, but not in AURKA-silenced cells exhibiting PD‐L1 overexpression.^142^ Consequently, AURKA inhibitors and cytokine-based immunotherapy may have a synergistic impact and provide a promising therapeutic approach for human malignancies.

## Conclusion and future prospect

Aurora Kinase A (AURKA) stands out as a pivotal regulator in cellular processes essential for mitosis and beyond, making it a significant player in cancer biology. The overexpression and dysregulation of AURKA in various cancers highlight its importance as both a diagnostic marker and a therapeutic target. Inhibition of AURKA has shown promise in preclinical and clinical studies, demonstrating efficacy in blocking tumor growth and overcoming therapeutic resistance. Beyond cancer, recent research has unveiled intriguing connections between AURKA and viral and bacterial infections, shedding light on its involvement in infectious diseases and their associated oncogenesis. Viruses such as Epstein-Barr virus and Kaposi’s Sarcoma-associated herpesvirus manipulate AURKA to promote cellular transformation, highlighting potential avenues for therapeutic intervention in virus-associated cancers. Similarly, bacterial infections like *H. pylori* and *S. aureus* influence AURKA expression and activity, linking chronic infection with increased cancer risk. Looking forward, further exploration of AURKA’s role in infection-induced cancers presents promising avenues for research and clinical application. Investigating the precise mechanisms by which viruses and bacteria manipulate AURKA could unveil new therapeutic targets and strategies. Targeting AURKA in combination with conventional cancer therapies or specific inhibitors tailored to infection-related pathways holds potential for enhancing treatment efficacy and overcoming resistance mechanisms. Moreover, elucidating the interplay between AURKA and the host immune response during infections may provide insights into immune evasion strategies employed by pathogens and opportunities for immune modulation in cancer therapy. Advances in understanding AURKA’s kinase-independent functions and its interactions with viral and bacterial proteins could lead to innovative approaches in precision medicine, aiming to tailor treatments based on infection status and molecular profiles. In conclusion, while AURKA continues to be a cornerstone in cancer research and therapy, its emerging role in infection-associated cancers opens up new vistas for combating these complex diseases. Integrating knowledge from infection biology with cancer biology promises to advance our understanding of disease mechanisms and accelerate the development of targeted therapies, ultimately improving patient outcomes and quality of life.

## Data Availability

Data sharing not applicable – no new data generated.
